# Experimental Evaluation of Performance of a Low-Initial-Viscosity Gel Flooding System

**DOI:** 10.3390/molecules29133077

**Published:** 2024-06-27

**Authors:** Cheng Fu, Bin Huang, Wei Zhang, Weisen Zhang, Shibo He

**Affiliations:** 1 College of Petroleum and Gas Engineering, Chongqing University of Science and Technology, Chongqing 401331, China; fu_cheng111@163.com; 2 Chongqing Institute of Unconventional Oil & Gas, Chongqing University of Science and Technology, Chongqing 401331, China; 3 Cnooc Energy Development Co., Ltd., Engineering Technology Branch, Tianjin 300450, China; 17606482650@163.com; 4 Sinopec Zhenhai Refining and Chemical Company, Ningbo 315221, China; heshibo1112@163.com

**Keywords:** low-initial-viscosity gel, polymer flooding, profile control

## Abstract

In order to effectively adjust reservoir heterogeneity and further exploit the remaining oil, a new type of low-viscosity gel was prepared by adding a regulating agent, retarder, and reinforcing agent on the basis of a polymer + Cr^3+^ crosslinking system. The new gel has the advantages of low initial viscosity, a slow gel formation rate, and high strength after gel formation. The effectiveness of the gel was verified through three-layer core displacement experiments, and the injection scheme was optimized by changing the slug combination of the polymer and the gel. The results showed that the gel can effectively block the high-permeability layer and adjust reservoir heterogeneity. An injection of 0.1 pore volume (PV) low-initial-viscosity gel can improve oil recovery by 5.10%. By changing the slug combination of the gel and polymer, oil recovery was further increased by 3.12% when using an injection of 0.07 PV low-initial-viscosity gel +0.2 PV high-concentration polymer +0.05 PV low-initial-viscosity gel +0.5 PV high-concentration polymer.

## 1. Introduction

Due to natural geological deposition and artificial development (including water injection, acid fracturing, etc.), reservoir fractures occur, and the water phase viscosity is lower than the oil phase viscosity, resulting in early water breakthrough, frequent water channeling, and other problems, which seriously hinder the stimulation effect of various oil fields [1,2,3,4,5]. As an effective method to improve reservoir heterogeneity and increase the swept volume, profile control has been widely used in oilfield development [6,7,8]. As a kind of plugging agent that can effectively improve the heterogeneity of a reservoir, gel is widely used in oilfields [9,10,11].

Many works about gel-plugging agents for enhanced oil recovery have been published in the past years. For example, hydrolytic polyacrylamide (HPAM)/Cr crosslinked gel systems, as a relatively common profile control agent, are widely used in many oil fields [12,13,14]. Wang et al. prepared a new type of high-temperature gel, which has good adaptability to temperature, good injectivity, and good erosion resistance [15]. Nie et al. investigated the plugging and profile control of polymer microspheres as a displacement fluid for enhancing oil recovery [16]. Sun et al. prepared a multifunctional gel by using phenyllactic acid, which reduced costs while ensuring the plugging effect [17]. Reihaneh Zolfaghari et al. developed a novel hydrogel by using partially hydrolyzed polyacrylamide, montmorillonite, and Cr and optimized its injection method [18]. Cui et al. studied an ultra-high molecular weight polymer gel synthesized by condensation reactions and optimized its parameters, which achieved good results in field application [19]. Hasankhani et al. used a mixture of HPAM, PEI, and asphaltene to prepare a novel polymer gel system with an enhanced oil recovery effect [20]. Alhuraishawy et al. improved recovery in high-carbonate reservoirs by combining low-salinity water flooding with prefabricated granular gels [21]. 

According to the core simulation experiment conducted by Sorbie et al., when the initial viscosity of a gel was greater than 20 mPa·s, the amount of polymer entering the medium- and low-permeability layers was 84% of that entering the high-permeability layer, which would cause a large degree of pollution to other permeability layers while blocking the high-permeability layer [22]. Therefore, under the premise of ensuring the strength of the gel, the injection viscosity of the gel should be reduced so that the gel can enter more thief zones to achieve effective plugging, adjust the heterogeneity of the reservoir, and increase the final recovery. The gels used in the oilfield usually have high viscosity, yet in the case of low viscosity, the gelling strength cannot be guaranteed. Especially for the Daqing Oilfield, the formation situation is relatively complex [23], and it is difficult for a general gel system to maximize oil recovery.

In order to solve this problem, a polymer gel was prepared by adding a regulating agent, retarder, and reinforcing agent on the basis of a polymer + Cr^3+^ crosslinking system. The gel has a lower initial viscosity and a slow gelatinization speed and has a strong gel-forming strength after the end of gelatinization. In order to verify the profile control effect of the gel, indoor oil displacement experiments of three layers of cores were carried out in this study. The effects of the low-initial-viscosity gel on adjusting reservoir heterogeneity and enhancing ultimate recovery were evaluated. By changing the injection method of low-initial-viscosity gel and high-concentration polymer, the best solution that can effectively improve reservoir heterogeneity and enhance oil recovery was obtained. The results of this study have strong reference significance for the field application of the gel.

## 2. Experiments

The preparation of the low-initial-viscosity gel-plugging control system involved restraining the rise in the water cut in the produced fluid and improving the recovery rate so as to realize the effective development of the oilfield. In order to verify the oil displacement performance of the gel-plugging control system in the actual reservoir, a three-layer core physical model was prepared to simulate the actual formation of oil displacement according to the geological characteristics of a block after polymer flooding in the Daqing oilfield; then, an oil displacement effect control experiment with or without the plugging agent, an injection time evaluation experiment of the gel-plugging control agent, and an injection slug optimization experiment were carried out. Through the detection of oil recovery, pressure, water content, and other indexes in different periods for each experiment, the oil displacement performance of the low-initial-viscosity gel-plugging control system was determined, which provides reliable data support for practical application in the field.

### 2.1. Materials

**Figure 1:**
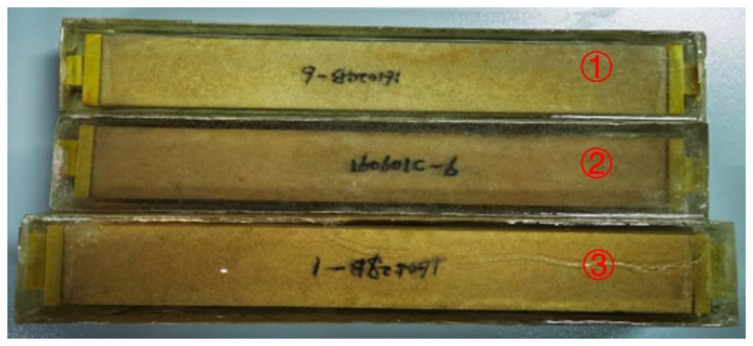
Three different types of artificial cores were used in this experiment (①: high permeability; ②: medium permeability; ③: low permeability).

The specific parameters of the experimental reagents used in this work are shown in Table 1. The experimental water used was the field reinjection water in the test block of the Daqing Oilfield, with a salinity of 5522 mg/L. Table 2 lists the ion concentration of the reinjection water. The mineralized water used for gel preparation had a salinity of 6778 mg/L, and the main components are shown in Table 3. The simulated crude oil was prepared from the crude oil and kerosene from the third production plant of the Daqing Oilfield in a certain proportion, with a viscosity of 9.8 mPa·s at 45 °C. The cores used in the experiment are all artificial cores cemented by quartz sand epoxy resin, which were divided into a high-permeability layer, medium-permeability layer, and low-permeability layer, as shown in Figure 1. The details of the parameters of these cores are listed in Table 4.

### 2.2. Experiment Apparatus

**Figure 2:**
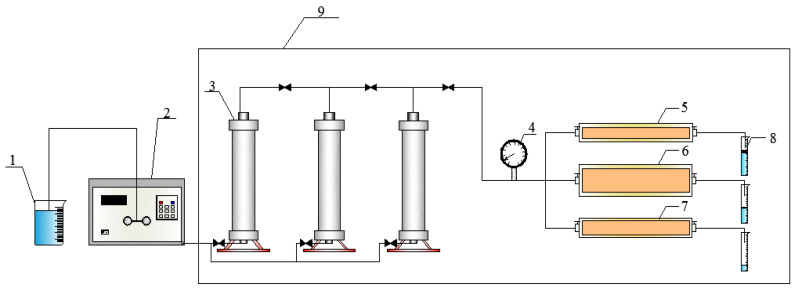
Schematic diagram of the three-layer core oil displacement experiment. 1—Beaker; 2—advection pump; 3—intermediate container; 4—pressure gauge; 5—high-permeability core; 6— medium-permeability core; 7—low-permeability core; 8—plastic pipelines; 9—constant temperature box.

The details of the parameters of the apparatus used in this work are listed in Table 5. Figure 2 shows the schematic diagram of the three-layer core oil displacement experiment.

### 2.3. Experiment Method

#### 2.3.1. Preparation of Polymers and Gels

According to the experimental design concentration, a certain amount of mineralized water and polymer dry powder were weighed, and the polymer dry powder was slowly added to the mineralized water under the condition of stirring at a certain speed, and this can be used after continuous stirring and curing for 3 h. Then, according to the concentration requirements of the experiment, a certain amount of retarder, enhancer, regulator, and crosslinking agent were weighed, respectively, and slowly added to the matured polymer solution in turn for mixing. After the stirring provided a uniform substance, the water bath temperature of the high-viscosity rheometer was set to 45 °C, the shear rate of the rotor was set to 4.51 s^−1^, and the initial viscosity of the gel-plugging control system was measured under this state. Then, it was put into a thermostatic box with an ambient temperature of 45 °C and taken out for viscosity measurement after a set period of time so as to record the low-viscosity period and the adhesive viscosity of the gel system. The concentrations of the components of the polymer gel are listed in Table 6.

#### 2.3.2. Experimental Procedure and Schemes

Details experimental steps are listed in Table 7.

The experimental scheme for the evaluation of the properties of the low-viscosity gel and injection slug optimization process are shown in Table 8.

The volume of produced fluid at the end of the core was measured to calculate the instantaneous shunt rate and recovery of each permeability layer. The time interval for each recording was 30 min. The injection rate of the water flooding stage was 1.2 mL/min, and the injection rate of the polymer flooding and gel flooding stage was 0.6 mL/min during the injection process.

## 3. Results and Discussions

### 3.1. Plugging Effect of Low-Initial-Viscosity Gel

#### 3.1.1. Instantaneous Shunt Rate

**Figure 3:**
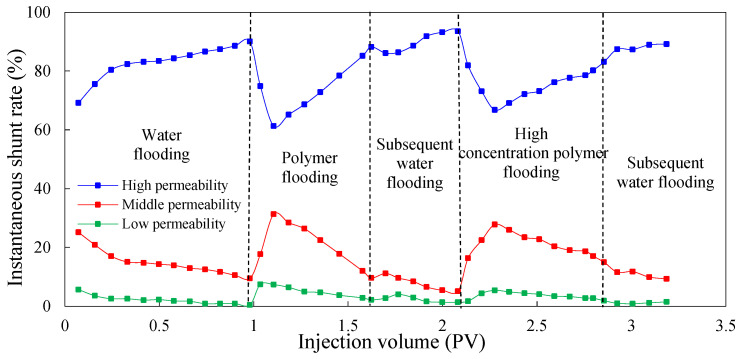
The instantaneous shunt rate for Scheme 1.

**Figure 4:**
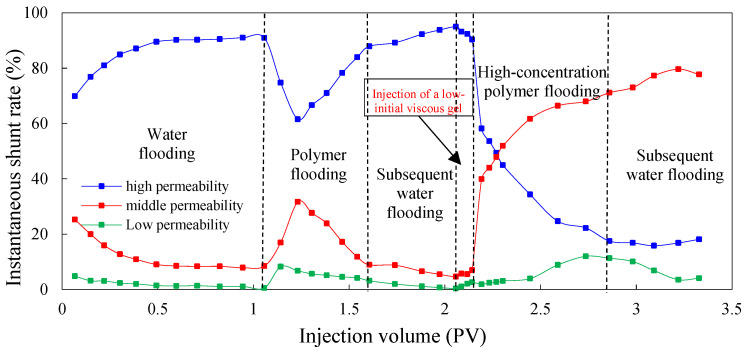
The instantaneous shunt rate for Scheme 2.

The instantaneous shunt rate can directly reflect the amount of liquid absorption of different permeable layers at each stage, and it can be used as a basis to judge the ability to adjust the water absorption profile to improve reservoir heterogeneity during the injection process of different agents. Figure 3 and Figure 4 show the instantaneous shunt rate in the experimental Schemes 1 and 2 under different injection processes, respectively.

As can be seen, in the process of water flooding, the amount of water injected into the high-permeability layer is obviously greater than that injected into the medium- and low-permeability layers. This is because the pore diameter of the high-permeability layer is large, and the percolating resistance is small compared to the medium- and low-permeability layers. With the increase in injection volume, the oil in the high-permeability layer is gradually recovered, and the percolating resistance is further reduced, which leads to the further enhancement of heterogeneity. Therefore, the liquid absorption of the high-permeability layer increases and the shunt rate becomes larger, whereas the liquid absorption of the medium- and low-permeability layers decreases and the shunt rate becomes smaller.

In the first process of polymer flooding, the liquid absorption of each layer is redistributed during the displacement process due to the polymer increasing the viscosity of the water phase. In the early stage of polymer flooding, the percolation resistance of the high-permeability layer is small, so the liquid absorption is relatively large. As the injection volume increases, the percolation resistance of the high-permeability layer will gradually increase, resulting in a gradual decrease in the instantaneous shunt rate, prompting subsequent polymers to enter the medium- and low-permeability layers with lower percolation resistance, and flow diversion occurs. However, due to the accumulation of the polymer in the medium- and low-permeability layers, the additional percolation resistance of the medium- and low-permeability layers is higher than that of the high-permeability layer, which leads to profile inversion in the middle and late stages of polymer flooding. The liquid absorption of the high-permeability layer increases and the shunt rate increases while the shunt rate of the medium- and low-permeability layers decreases gradually. In the stage of subsequent water flooding, due to the poor retention ability of polymer in the core and the limited ability of profile adjustment in the reservoir, the percolation resistance of the high-permeability layer decreases, and the shunt rate increases gradually. Meanwhile, the shunt rate of the medium- and low-permeability layers decreases gradually. After subsequent water flooding, the shunt rate of the high-permeability layer increases to 93.56%, and that of the medium- and low-permeability layers decreases to 5.10% and 1.34%.

In the chemical flooding process, the regularity of the shunt rate in different experimental Schemes was very different. Because the low-initial-viscosity gel was not injected in the chemical flooding process of Scheme 1 for profile control, the shunt rate of the high-permeability layer gradually decreased and then increased after the injection of high-concentration polymer. This is because the viscosity of the high-concentration polymer is higher than water, meaning the seepage resistance of the high-permeability layer increased, forcing the liquid to flow to the medium- and low-permeability layers. However, with the increase in the injected amount of high-concentration polymer, the additional seepage resistance of the medium- and low-permeability layers gradually exceeded the seepage resistance of the high-permeability layer, and profile inversion occurred again. Therefore, the shunt rate of the high-permeability layer increased, and the shunt rate of the medium- and low-permeability layers decreased. In Scheme 2, the shunt rate of the high-permeability layer continued to decrease, whereas the shunt rate of the medium- and low-permeability layers continued to increase. This indicates that the injection of low-initial-viscosity gel increased the seepage resistance of the high-permeability layer, leading to the shunt rate of the high-permeability layer to continually decrease and the shunt rate of the medium- and low-permeability layers continued to increase. This proves that the low-initial-viscosity gel can effectively block the high-permeability layer and has a strong control ability profile.

#### 3.1.2. Dynamic Parameters

**Figure 5:**
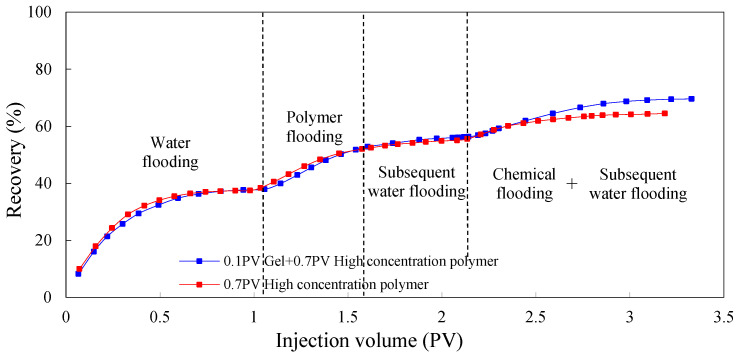
EOR comparison between Schemes 1 and 2.

**Figure 6:**
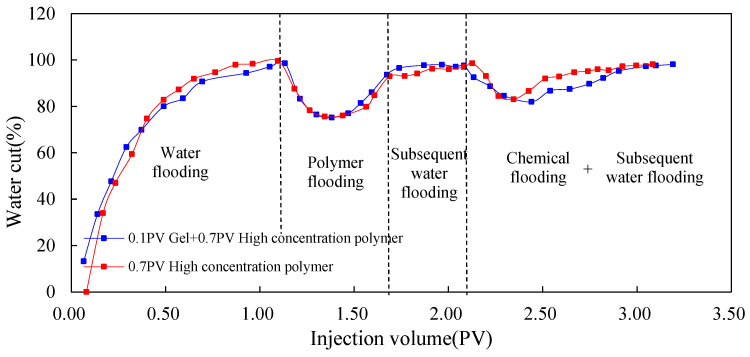
Water cut comparison between Schemes 1 and 2.

**Figure 7:**
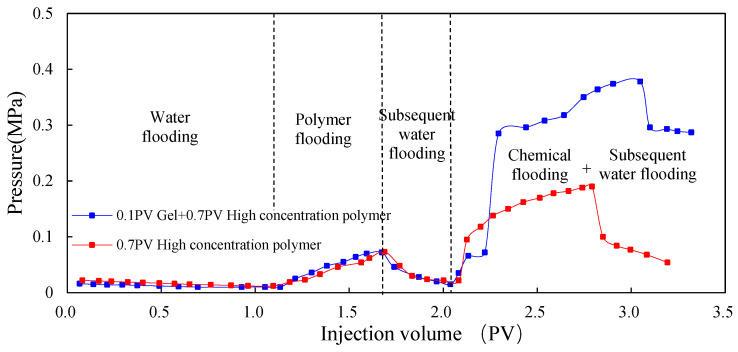
Pressure comparison between Schemes 1 and 2.

Figure 5, Figure 6 and Figure 7 show the dynamic parameter changes of Scheme 1 and 2. The profile control ability of the gel was evaluated in terms of three aspects: recovery factor, water content, and injection pressure. By comparison, during the chemical flooding process, the seepage resistance of Scheme 2 significantly increased due to the use of low-initial-viscosity gel for profile control, and the pressure of Scheme 2 significantly increased compared to that of Scheme 1. At the same time, due to the effective plugging of the high-permeability layer, the water cut during the chemical flooding process significantly decreased, and the ultimate recovery factor significantly increased. The recovery of Scheme 2 was 5.10 percentage points higher than that of Scheme 1.

### 3.2. Optimization Experiment of Injection Mode of Oil Displacement System

The injection method of 0.1 PV low-initial-viscosity gel + 0.7 PV high-concentration polymer can effectively improve oil recovery, but the effect of improving oil recovery and reducing the water cut is limited. Therefore, slug optimization experiments were undertaken to screen for the experimental scheme that could improve oil recovery and reduce the water cut to the greatest extent.

#### 3.2.1. Instantaneous Shunt Rate

**Figure 8:**
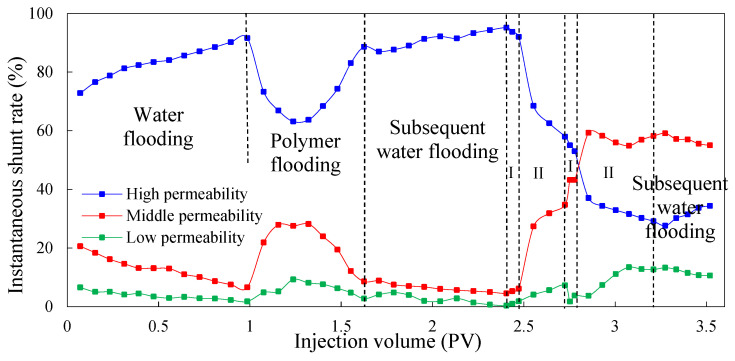
The shunt rate of each permeable layer in Scheme 3.

**Figure 9:**
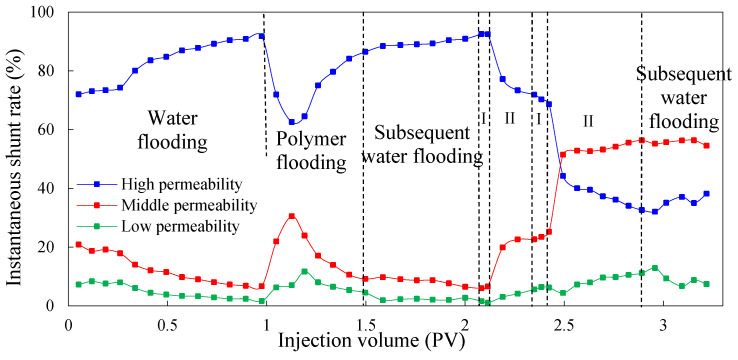
The shunt rate of each permeable layer in Scheme 4.

**Figure 10:**
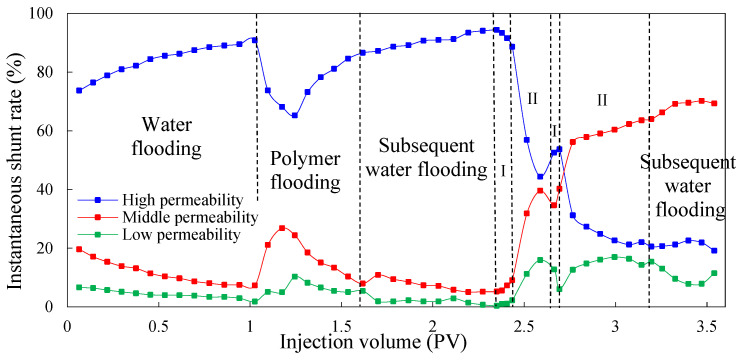
The shunt rate of each permeable layer in Scheme 5.

When double-round profile control is adopted, Figure 8, Figure 9 and Figure 10 show the variation in the shunt rate of each permeable layer at different processes. The injection processes of the low-initial-viscosity gel and high-concentration polymer are indicated as I and II in the figures, respectively. In the process of water flooding and polymer flooding before profile control via gel, the regularity of each experimental scheme is the same; it will not be repeated here. Instead, we mainly analyzed the regularity of the shunt rate of each permeability layer after profile control by injecting the low-initial-viscosity gel. In the primary profile control process, due to the high viscosity of the gel, the amount of liquid was redistributed among the layers, and the shunt rate of the high-permeability layer decreased, with the shunt rate of the medium- and low-permeability layers increasing. Because the total liquid absorption amount of the high-permeability layer was far greater than that of the medium- and low-permeability layers, the effective plugging of the high-permeability layer could be realized after gelatinization. In the first process of high-concentration polymer injection, the high-permeability layer was effectively blocked due to the injection of the low-initial-viscosity gel blocking agent. The shunt rate of the high-permeability layer continued to decrease, and the shunt rate of the medium- and low-permeability layers continued to increase. When compared to Schemes 3 and 4, and due to the larger volume of the front low-initial-viscosity gel slug in Scheme 5, the plugging effect on the high-permeability layer is better, and more high-concentration polymer enters the medium-permeability layer. However, as the accumulation of polymers leads to an increase in the additional percolation resistance of the medium-permeability layer, more oil-displacing agents enter the high-permeability layer, the shunt rate of the high-permeability layer increases, and the shunt rate of the medium-permeability layer decreases.

During the secondary plugging process using the low-initial-viscosity gel, because the heterogeneity of the reservoir was adjusted by the gel and high-concentration polymer, the low-initial-viscosity gel could penetrate more into the medium-permeability layer. It has a certain blocking effect on the medium-permeability layer, playing a certain role in plugging the medium-permeability layer so as to improve the production degree of the low-permeability layer in the subsequent displacement process. Additionally, at the stage of the secondary injection of the high-concentration polymer, the high-permeability layer is effectively blocked, and the shunt rate continues to decline. The shunt rate of the medium and low-permeability layers increases. When compared to the single-round blocking adjustment, the double-round profile control process develops the low-permeability layer to a greater extent. By comprehensively comparing the effects of the three groups of experiments in terms of adjusting the shunt rate, the injection method of Scheme 5 can minimize the shunt rate of the high-permeability layer and improve the shunt rate of the low-permeability layer. The effect of the three groups of experiments on adjusting the shunt rate is shown in Table 9. After the subsequent water flooding, the shunt rate of the high-permeability layer in experimental Scheme 5 decreased to 19.16%, and the shunt rate of the medium-permeability layer and low-permeability layer increased to 69.34% and 11.50%, respectively.

#### 3.2.2. Dynamic Parameters

**Figure 11:**
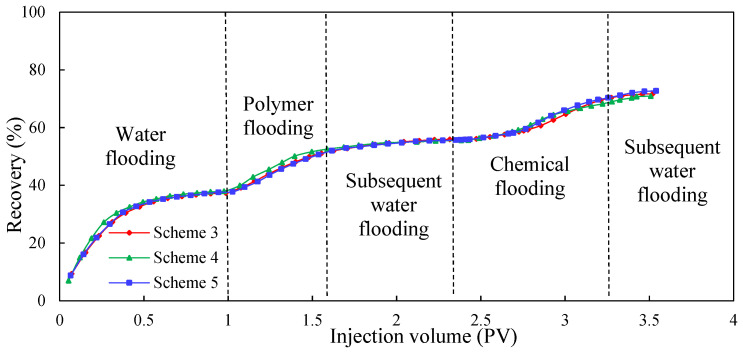
EOR comparison of Schemes 3–5.

**Figure 12:**
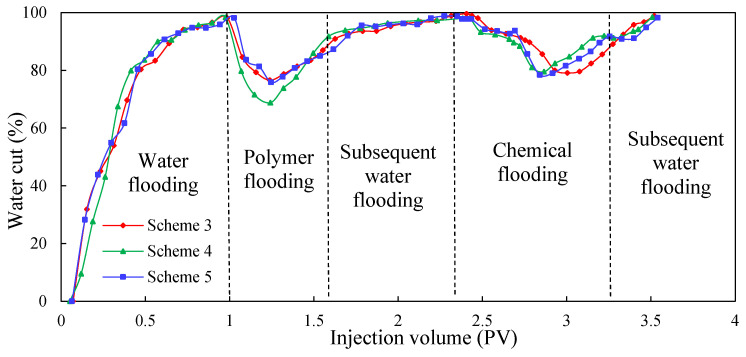
Water cut comparison of Schemes 3–5.

**Figure 13:**
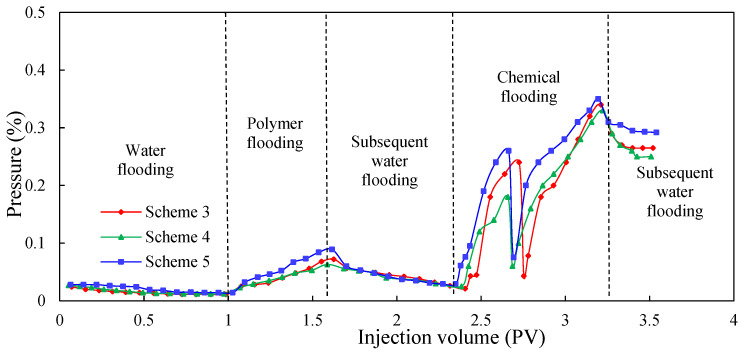
Pressure comparison of Schemes 3–5.

Figure 11, Figure 12 and Figure 13 show the dynamic parameter comparison curves of each group in different stages of the experimental schemes. The total injection amount of low-initial-viscosity gel and high-concentration polymer remains the same: the injection method of multiple slug combination can reduce the water cut to a greater extent and improve oil recovery. Among them, after the subsequent water flooding after polymer flooding, the injection method of Scheme 5 is the best. The Scheme 2 injection method (0.1 PV low-initial-viscosity gel-plugging agent injection) can effectively plug the high-permeability layer, but in the subsequent 0.7 PV high-concentration polymer injection process, the shunt rate of the low-permeability layer increases slowly, and more oil displacement agents flow into the medium-permeability layer with small percolation resistance, leading to poor development effects of the low-permeability layer; therefore, the degree of reduction of the comprehensive water content is poor. For the slug injection method, the lead plugging agent slug can realize the effective plugging of the high-permeability layer. In the subsequent injection process using the 0.2 PV high-concentration polymer, the additional percolation resistance of the high-permeability layer increases, which is conducive to the subsequent plugging agent entering into the medium-permeability layer more to achieve a certain degree of plugging of the medium-permeability layer; this is conducive to the development of the low-permeability layer. In this process, the percolation resistance increases greatly, the water cut decreases, and the recovery further increases.

When compared to Schemes 3 and 4, Scheme 5 has the best effect on reducing the water cut and enhancing oil recovery. Injecting a large slug with 0.07 PV can achieve a strong plug over the high-permeability layer. In the process of 0.2 PV high-concentration polymer flooding, the injection pressure increases, and the water cut decreases significantly, with recovery increasing greatly. During the subsequent injection of the 0.03 PV plugging agent slug, the medium-permeability zone was blocked to a certain extent, and the low-permeability layer was activated more, meaning the injection pressure increased, the water cut decreased, and recovery further improved. During this process, the injection pressure can reach as high as 0.36 MPa, the water cut can be as low as 78.42%, and recovery is greatly increased. The final recovery reached 72.71%, which is 1.29 and 1.41 percentage points higher than the recovery factor of Schemes 3 and 4, respectively.

According to Table 10, it can be seen (by comparison) that under the premise of a certain amount of low-initial-viscosity gel and high-concentration polymer injection, the two-round profile control injection method has a better effect on EOR than the single-round profile control injection method. In addition, the plugging adjustment method of preferentially injecting large slugs of low-initial-viscosity gel and then injecting small slugs of low-initial-viscosity gel is more conducive to improving oil recovery.

## 4. Conclusions

(1) The low-initial-viscosity gel can effectively plug the high-permeability layer, expand the swept volume of the subsequent oil displacement system, and improve formation heterogeneity. After an injection of 0.12 PV low-viscosity retarded gel, the flow rate of the high-permeability layer decreased from 93.04% to 23.17%, and the flow rate of the medium- and low-permeability layers increased by 73.70% and 3.13%, respectively. The final oil recovery was 3.30% higher than that of using high-concentration polymer injection only.
(2) Under the premise that the amount of injected low-viscosity retarding gel and high-concentration polymer is certain, the injection method of 0.08 PV low-viscosity retarding gel + 0.2 PV high-concentration polymer + 0.04 PV low-viscosity retarding gel + 0.6 PV high-concentration polymer can improve bottom-layer heterogeneity to the greatest extent and effectively block the dominant channel. The fractional flow rate of the high-permeability layer decreased to 19.2%, and the fractional flow rate of the medium- and low-permeability layers increased to 69.3% and 11.5%, respectively. The final oil recovery reached 72.71%, which was 4.70% higher than that of the injection method of 0.12 PV low-viscosity slow coagulation gel + 0.8 PV high-concentration polymer.

## Figures and Tables

**Table 1:** Specific parameters of the experimental reagents.

Reagent Name	Parameters	Source
Polymer LH2500	relative molecular weight: 2500 × 10^4^, degree of hydrolysis: 25%, solid content: 90%, industrial products	Petrochina Daqing Refining and Chemical Company (Daqing, China)
Metal ion chelating crosslinking agent CYJL	effective ion content: 2.5%, industrial product	Daqing Irradiation Center (Daqing, China)
Regulating agent	citric acid effective content: 99.8%, analytical pure	Shanghai United Test chemical reagent Co., Ltd. (Shanghai, China)
Retarder	sodium sulfite effective content: 99%, analytical pure	Hebei Mojin Biotechnology Co., Ltd. (Shijiazhuang, China)
Reinforcing agent	sodium polyphosphate effective content: 99.5%, analytical pure	Shandong Tengwang Chemical Co., Ltd. (Zaozhuang, China)

**Table 2:** Ion concentration of field reinjection water in Daqing Oilfield.

An Ion	Ca^2+^	Mg^2+^	HCO_3_^−^	CO_3_^2−^	Cl^−^	K^+^ + Na^+^	SO_4_^2−^
Ionic salinity (mg/L)	36.11	20.61	2019.03	589.58	1032.45	1809.41	15.27

**Table 3:** Main components of mineralized water.

Component	NaCl	KCl	CaCl_2_	Na_2_SO_4_	NaHCO_3_	MgSO_4_
Concentration (mg/L)	3489	20	64	114	2829	262

**Table 4:** Details of the parameters of three different types of cores.

Core Types	High-Permeability Layer	Medium-Permeability Layer	Low-Permeability Layer
Core specification (mm)	300 × 45 × 18	300 × 45 × 45	300 × 45 × 20
Air permeability (μm^2^)	4.0	2.0	0.5

**Table 5:** Details of the parameters of the experiment apparatus.

Name	Type	Source
Advection pump	2PB00C	Beijing Star Technology Development Co. Ltd. (Beijing, China)
Electronic scale	D2004W	Shanghai Sile Instrument Co., Ltd. (Shanghai, China)
Intermediate container	ZR-3	Hai’an Petroleum Instrument Co., Ltd. (Hainan, China)
High-viscosity rheometer	AR2000EX TA	Waters Technology (Shanghai) Co., Ltd. (Shanghai, China)
Vacuum pump	2XZ-4	Hai’an Petroleum Instrument Co., Ltd. (Hainan, China)
Constant temperature box	GTL-1	Nantong Zhongjing Machinery Co., Ltd. (Nantong, China)

**Table 6:** The concentration of the component of the polymer gel.

Agent	Crosslinking Agent	Citric Acid	Sodium Sulfite	Sodium Polyphosphate
Concentration (mg/L)	1500	300	150	150

**Table 7:** Experimental steps.

No.	Experimental Steps
1	At room temperature, cores with different permeabilities were vacuumed for 8 h before their dry weight was measured. After saturation with formation water for 12 h, the wet weight was measured, and the core pore volume was calculated.
2	The core was placed in an incubator at 45 °C and saturated oil to the end until no water was produced; it was aged at constant temperature for 12 h to calculate the oil saturation of the core in different permeable layers.
3	Water flooding was carried out in an incubator at 45 °C to measure the liquid output at the end of the core. After the water content reached 98% and 0.57 PV, polymer flooding was carried out.
4	The chemical agent was injected into the incubator at 45 °C, according to the experimental scheme.
5	Subsequent water flooding was carried out to measure the liquid output at the end of the core. The displacement experiment was stopped when the water cut reached 98%.

**Table 8:** Experimental schemes.

Name	Number	Scheme
Evaluation of low-initial-viscosity gel	1-1	0.7 PV high-concentration polymer flooding
	1-2	0.1 PV low-initial-viscosity gel for profile control + 0.7 PV high-concentration polymer flooding
Optimization of injection slug	2-1	0.05 PV low-initial-viscosity gel for profile control + 0.2 PV high-concentration polymer flooding + 0.05 PV low-initial-viscosity gel for profile control + 0.5 PV high-concentration polymer flooding
	2-2	0.03 PV low-initial-viscosity gel for profile control +0.2 PV high-concentration polymer flooding + 0.07 PV low-initial-viscosity gel for profile control +0.5 PV high-concentration polymer flooding
	2-3	0.07 PV low-initial-viscosity gel for profile control +0.2 PV high-concentration polymer flooding + 0.03 PV low-initial-viscosity gel for profile control +0.5 PV high-concentration polymer flooding

**Table 9:** The shunt rate before and after plugging adjustment in different experimental schemes.

Experimental Scheme	Core Type	Shunt Rate before Blocking Adjustment (%)	Shunt Rate after Blocking Adjustment (%)
Scheme 3	High-permeability layer	94.33	38.38
	Middle-permeability layer	4.97	55.01
	Low-permeability layer	0.70	10.61
Scheme 4	High-permeability layer	94.07	38.15
	Middle-permeability layer	4.90	54.50
	Low-permeability layer	1.03	7.36
Scheme 5	High-permeability layer	94.36	19.16
	Middle-permeability layer	5.24	69.34
	Low-permeability layer	0.39	11.50

**Table 10:** EOR in different stages for different injection methods and experimental schemes.

	Scheme 3	Scheme 4	Scheme 5
Polymer flooding recovery	56.12	55.98	55.95
Ultimate recovery factor	71.16	70.64	72.11
EOR enhancement value	15.04	14.66	16.16

## Data Availability

The data presented in this study are available in article.
